# The effect of surgeon volume on outcomes after total hip arthroplasty: a systematic review and meta-analysis

**DOI:** 10.1007/s00402-026-06434-w

**Published:** 2026-08-20

**Authors:** Nils Meissner, Katharina Ortwig, Sonia Ramos-Pascual, Chinyelum Agu, Johannes Stoeve, Daniel Schrednitzki, Tim Rolvien, Andreas M. Halder

**Affiliations:** 1 Department of Orthopaedic Surgery, Sana Hospital Sommerfeld, Kremmen, Germany; 2 German Society for Orthopedics and Trauma Surgery, Berlin, Germany; 3 ReSurg (Switzerland), Nyon, Switzerland; 4 Department of Orthopaedic and Trauma Surgery, St. Marien- und St. Annastiftskrankenhaus, Ludwigshafen am Rhein, Germany; 5 Department of Orthopaedic, Trauma, Hand and Reconstructive Surgery, Sana Klinikum Lichtenberg, Berlin, Germany; 6 Department of Trauma and Orthopaedic Surgery, Division of Orthopaedics, University Medical Center Hamburg-Eppendorf, Hamburg, Germany

**Keywords:** Systematic review, Meta-analysis, Surgeon volume, Provider volume, Total hip arthroplasty

## Abstract

**Purpose:**

To synthesize, critically appraise and systematically review studies comparing outcomes after primary total hip arthroplasty (THA) performed by low- vs. high-volume surgeons, and to propose evidence-based threshold definitions for these categories.

**Methods:**

This review followed the PRISMA guidelines and was registered in PROSPERO. Medline and Embase were searched on March 24, 2025. Prospective and retrospective clinical studies were included if they reported THA thresholds for low- and high-volume surgeons. Articles not written in English or German were excluded. Outcomes were pooled and presented in forest plots. Methodological quality was assessed.

**Results:**

Of 872 identified articles, 18 were included, reporting on 796,061 patients undergoing THA. Methodological quality was high in all studies (>6 of 7 points). Thresholds used to define high- vs. low-volume were determined using arbitrary values (n = 11), tertiles (n = 1), quartiles (n = 3), percentiles (n = 2), or receiver operating characteristic curves (n = 1). High-volume surgeons had significantly lower 1-month readmission rates [odds ratio (OR) = 1.6; *p* = 0.033] and 3-month mortality rates (OR = 1.8; *p* = 0.008). In contrast, there were no significant differences between high- and low-volume surgeons for 3-month revision rates (OR = 2.0; *p* = 0.228), 12-month revision rates (OR = 1.8; *p* = 0.228), and 3-month readmission rates (OR = 1.3; *p* = 0.337).

**Conclusions:**

The included studies demonstrated considerable variability in the definitions of surgeon volume. However, there remains a trend suggesting that higher-volume surgeons experience fewer revisions, complications, readmissions, and mortality; although these differences were largely not statistically significant. Nonetheless, surgeon volume may not be the only factor influencing outcomes, as hospital and patient characteristics may influence short- to long-term outcomes following primary THA.

**Supplementary Information:**

The online version contains supplementary material available at 10.1007/s00402-026-06434-w.

## Introduction

Total hip arthroplasty (THA) is a cost-effective treatment for hip osteoarthritis, that has demonstrated high survival rates and excellent clinical outcomes [16, 28, 52]. Numerous studies have investigated the effect of surgeon volume on THA outcomes, with contradictory findings [6, 56, 59, 75, 76, 83].

A systematic review [44] from 2018 that included articles reporting on patients undergoing surgery for hip fractures, found that higher caseloads were associated with fewer complications and improved recovery rates, compared to surgeons that performed less yearly caseloads, although no significant association with clinical scores was observed. Conversely, Murphy et al. [56] found that low-volume surgeons occasionally outperformed high-volume surgeons, whereas more recent clinical studies have demonstrated no consistent associations between caseload and postoperative outcomes [59, 76].

In addition, definitions of “low-” and “high-” volume surgeons vary substantially across the literature [30, 36, 56]. Reported thresholds for high-volume surgeons range from as few as 15 THAs per year [11] to 150 THAs per year [68], complicating direct comparisons across studies. Consequently, the association between surgeon caseload and THA outcomes may largely depend on the applied volume thresholds. As such, the purpose of this meta-analysis was to synthesize, critically appraise and systematically review studies comparing primary THA outcomes performed by low- vs. high-volume surgeons, with particular emphasis on revisions, complications, readmissions, mortality, and clinical scores, and to propose evidence-based threshold definitions for these categories.

## Materials and methods

This systematic review followed the Preferred Reporting Items for Systematic Reviews and Meta-Analyses (PRISMA) guidelines and was registered with PROSPERO (CRD420251018348). There were no deviations from the protocol registered in PROSPERO. An electronic search of Medline (PubMed) and Embase was conducted on March 24, 2025 using predefined keywords (Appendix 1).

### Study selection

After removing duplicates, two reviewers (KO and CA) independently screened titles and abstracts in Rayyan [63] (Cambridge, Massachusetts, USA) to assess eligibility. The same two reviewers then assessed full texts independently. Articles were included if they were prospective or retrospective clinical studies reporting THA thresholds for low- and high-volume surgeons, and/or comparing outcomes following primary THA in low- vs. high-volume surgeons. Both randomised controlled trials (RCTs) and non-randomised studies of intervention (NRSIs) were included in the present review, as they can provide direct outcome comparisons. Outcomes of interest were complications, readmissions, revisions, mortality, and all clinical scores. Articles were excluded if they (i) reported on combined results for THAs and total knee arthroplasties (TKAs), where neither thresholds nor outcomes were presented separately for each surgery; (ii) were non-comparative studies or case series that did not report surgeon volume thresholds; (iii) were case reports, reviews, editorials, expert opinions, letters to the editor, or conference proceedings; (iv) reported on cadavers, animals, in-vitro experiments, or in-silico simulations; or (v) were written in languages other than English or German, to avoid translation errors. Experts in the field were asked to provide any further studies (not selected during screening) that they thought relevant.

### Data extraction and quality assessment

Data extraction was performed independently by two reviewers (CA and AA). Study and cohort characteristics, definition and thresholds of surgeon volume, and outcomes of interest were extracted. Methodological quality was assessed independently by two reviewers (CA and AA) using the Mixed Methods Appraisal Tool (MMAT) [25, 26]. Discrepancies during full text screening, data extraction and methodological quality assessment were resolved through review and consensus, or by involving a third reviewer (SRP).

### Data analyses

Categorical outcomes were reported as events and proportions, while continuous outcomes were reported as means, standard deviations, and ranges. Data on revisions, venous thromboembolisms, readmissions, and mortality were pooled and illustrated using forest plots, and where appropriate, data was stratified by follow-up. If an article categorized surgeon volume into more than two categories (e.g. low, moderate and high), we regrouped the patients to only have two categories (high vs. low) by combining groups. Pooled estimates of proportions, odds ratios (OR) and their 95% confidence intervals (95%CI) were calculated using the Freeman-Tukey double arcsine transformation and inverse-variance weighting within a frequentist random-effects model framework. A random-effects model was selected because it incorporates both within-study variance and between-study heterogeneity, providing more appropriate weighting when true effects may vary across studies. Between-study variance was estimated using the DerSimonian–Laird method, while Hartung–Knapp adjustment was not applied. Heterogeneity was evaluated by visual inspection of forest plots and by calculating the I^2^ statistic, which quantifies the proportion of total variability attributable to heterogeneity rather than sampling error, and its associated χ^2^ test [24]. A sensitivity analysis was performed including studies that defined high-volume surgeons as ≥50 THAs per year, and the ORs and corresponding 95% CI were calculated for outcomes that were reported in two or more studies (Appendix 2). Funnel plots were not performed because each meta-analysis contained fewer than ten studies. With such small numbers, funnel plots and associated tests can have low power and produce misleading conclusions about publication bias. *p*-values < 0.05 were considered statistically significant. Statistical analyses were performed using R version 4.5.0 (R Foundation for Statistical Computing, Vienna, Austria).

## Results

### Literature search

**Fig. 1:**
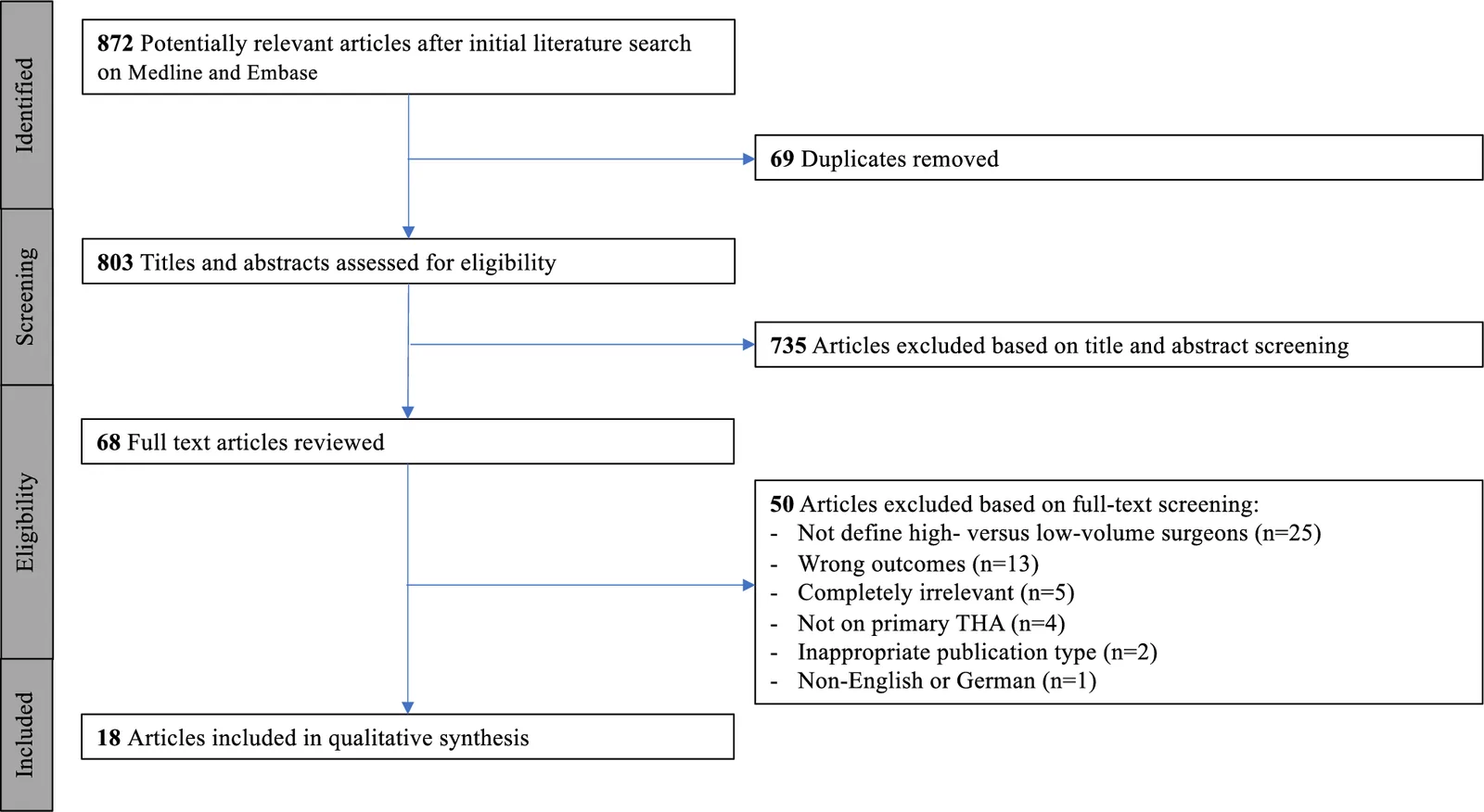
Flowchart presenting included and excluded articles

The systematic search returned 872 records, of which 69 were duplicates, leaving 803 for screening (Fig. 1). A total of 735 studies were excluded by examining their titles and/or abstracts, and a further 50 studies were excluded after full-text review for the following reasons: 25 did not define high- vs. low-volume surgeons [3, 9, 14, 15, 20, 21, 32, 33, 35, 36, 38, 40, 41, 43, 45, 46, 48, 49, 58, 59, 64, 67, 74, 75, 77], 13 reported the wrong outcomes [5, 10, 23, 29–31, 34, 39, 53, 54, 61, 69, 71], five were completely irrelevant [4, 7, 12, 19, 57], four were not on primary THA [47, 60, 81, 84], two were an inappropriate publication type [18, 79], and one was written in a language other than English or German [62]. No further studies were identified through reference list screening, or recommended by experts in the field, resulting in the inclusion of 18 studies [2, 3, 6, 8, 11, 13, 22, 27, 37, 42, 56, 65, 66, 68, 70, 72, 78, 80], on a total of 887,718 THAs (Table 1). There were six studies that disclosed conflicts of interest, and six declared external funding (Appendix 3). Eight studies were conducted in the United States, three in Canada, two each in the United Kingdom and Taiwan, and one each in France, Australia, and Belgium. Methodological quality was high, with 14 studies scoring seven of seven points and four scoring six of seven using the MMAT (Table 2).

**Table 1:** Characteristics of the included articles

First author, year	Study type	Journal	Country	Funding	COI	Database	Inclusion period for THA	Total N patients	Indication for THA	Definition of surgeon volume
Thomas, 2024 [80]	Retrospective	Bone and Joint Journal	USA	No	Yes	Urban tertiary health system	2013–2021	567 (matched cohort)	NR	High volume: (Top quartile) 173.7 ± 116.1 THAs/year (mean) Low volume: (Bottom 3 quartiles) 42.8 ± 27.4 THAs/year (mean)
Ruangsomboon, 2024 [72]	Retrospective	Acta Orthopaedica	Canada	No	No	Institute for Clinical Evaluative Sciences	2016–2021	9672	OA	High volume: >60 THAs/year Medium volume: 30–60 THAs/year Low volume: <30 THAs/year
Rajahraman, 2023 [68]	Retrospective	Arthroplasty Today	USA	NR	Yes	Urban tertiary academic health system	2012–2020	643	OA, DDH, RA, PTA, AVN, other	High volume: (Top quartile)150.5 THAs/year Low volume: (Bottom 3 quartiles) 38.5 THAs/year
Brodeur, 2022 [6]	Retrospective	The Journal of Arthroplasty	USA	NR	No	New York Statewide Planning and Research Cooperative System	2009–2014	45,299	OA	High volume: ≥115 THAs/year Low volume: ≤20 THAs/year
Chou, 2019 [11]	Retrospective	The Journal of Arthroplasty	Taiwan	Yes	No	Nationa research database	2012	6367	OA and others	High volume: ≥15 THAs/year Low volume: <15 THAs/year
Hanly, 2019 [22]	Observational	J Formos Med Assoc	Australia	No	Yes	Australian Orthopaedic Association National Joint Replacement Registry	2003–2016	13,051	OA	High volume: >25 THAs/year Medium volume: 10–25 THAs/year Low volume: ≤10 THAs/year
Murphy, 2019 [56]	Retrospective	Clinical Orthopaedics and Related Research	USA	Yes	Yes	Centers for Medicare & Medicaid Services Limited Data Set	2013–2016	4,09,844	NR	Highest volume: >50 THAs/year High volume: 26–50 THAs/year Moderate volume: 11–25 THAs/year Low volume: 6–10 THAs/year Lowest volume: 1–5 THAs/year
Annan, 2017 [2]	Prospective	European Journal of Orthopaedic Surgery & Traumatology	UK	NR	Yes	Hospital database	1997–2011	3744	OA, RA, other	The threshold values were assigned using the maximum Youden (J) Index calculated from receiver operating characteristic (ROC) curves
Le Cossec, 2017 [13]	Retrospective	Arthroplasty Today	France	NR	No	French National Health Insurance Information System	2010–2011	62,906	NR	High volume: (1st tertile), >4 THAs/month Medium volume: (2nd tertile), 1.5–4 THAs/month Low volume: (3rd tertile), <1.5 THAs/month
Paxton, 2015 [66]	Retrospective	Clinical Orthopaedics and Related Research	USA	No	No	Total Joint Replacement Registry	2009–2011	12,030	OA	High volume: ≥50 THAs/year Medium volume: 20–49 THAs/year Low volume: <20 THAs/year
Ravi, 2014 [70]	Propensity score matched cohort study	BMJ	Canada	Yes	No	Health administrative databases from Ontario, Canada (CIHI-DAD, OHIP, OPHRDC, and CPDB)	2002–2009	6716 (matched cohort)	OA	High volume: >35 THAs/year Low volume: <35 THAs/year
Baker, 2011	Retrospective	Journal of the Royal Colleges of Surgeons of Edinburgh and Ireland	UK	NR	No	Hospital database	2006–2007	248	OA, RA, other	High volume: >52 THAs/year Low volume: ≤52 THAs/year
Camberlin, 2011 [8]	Retrospective	Acta Orthopædica Belgica	Belgium	No	NR	Belgian hospital discharge administrative database	2004	11,856	OA, other	High volume: >20 THAs/year Medium volume: 7–20 THAs/year Low volume: ≤6 THAs/year
Huang, 2011 [27]	Retrospective	J Formos Med Assoc	Taiwan	NR	NR	National Health Insurance Research Databases	2005–2006	9335	AVN, OA, RA, other	High volume: (>80th percentile), 8–119 THAs/year Medium volume: (40–80th percentile), 2–7.5 THAs/year Low volume: (<40th percentile), 0.5–1.5 THAs/year
Styron, 2011 [78]	Retrospective	J Arthroplasty	USA	NR	NR	Health Care Utilization Project-Nationwide Inpatient Sample	NR	1,92,554	NR	Highest volume: (1st quartile), ≥53 THAs/year High volume: (2nd quartile), 22–52 THAs/year Moderate volume: (3rd quartile), 11–21 THAs/year Low volume: 1–10 (4th quartile), THAs/year
Paterson, 2010 [65]	Retrospective	Can J Surg	Canada	Yes	Yes	Institute for Clinical Evaluative Sciences	2000–2004	20,290	RA, other	Highest volume: >60 THAs/year High volume: 41–60 THAs/year Moderate volume: 26–40 THAs/year Low volume: 2–25 THAs/year
Losina, 2004 [42]	Retrospective	Arthritis and Rheumatism	USA	Yes	NR	National sample of US Medicare beneficiaries	1995–1996	57,488	OA, AVN, RA, other	High volume: >12 THAs/year Low volume: < 12 THAs/year
Kreder, 1997 [37]	Retrospective	The Journal of Bone & Joint Surgery	USA	Yes	No	Washington State Department of Health	1988–1991	7936	OA, RA, AVN	High volume: (> 80th percentile), > 10 THAs/year Medium volume: (40–80th percentile), 2–10 THAs/year Low volume: (<40th percentile), <2 THAs/year)

*THA* total hip arthroplasty, *OA* osteoarthritis, *RA* rheumatoid arthritis, *DDH* developmental dysplasia of hip, *PTA* posttraumatic arthritis, *AVN* avascular necrosis, *NR* not reported, *DDA* direct anterior approach

**Table 2:** Mixed methods appraisal tool (MMAT) for quantitative non-randomized studies

First author, year	Quantitative non-randomized							Quantitative descriptive					
	1.1. Are there clear research questions?	1.2. Do the collected data allow to address the research questions?	2.1. Are the participants representative of the target population?	2.2. Are measurements appropriate regarding both the outcome and intervention (or exposure)?	2.3. Are there complete outcome data?	2.4. Are the confounders accounted for in the design and analysis?	2.5 During the study period, is the intervention administered (or exposure occurred) as intended?	3.1. Is the sampling strategy relevant to address the research question?	3.2. Is the sample representative of the target population?	3.3. Are the measurements appropriate?	3.4. Is the risk of nonresponse bias low?	3.5. Is the statistical analysis appropriate to answer the research question?	Total score (max. 7)
Thomas, 2024 [80]	Yes	Yes						Yes	Yes	Yes	Yes	Yes	7
Ruangsomboon, 2024 [72]	Yes	Yes						Yes	Yes	Yes	Yes	Yes	7
Rajahraman, 2023 [68]	Yes	Yes						Can’t tell	Yes	Yes	Yes	Yes	6
Brodeur, 2022 [6]	Yes	Yes	Yes	Yes	Yes	Yes	Yes						7
Chou, 2019 [11]	Yes	Yes	Yes	Yes	Yes	Yes	Yes						7
Hanly, 2019 [22]	Yes	Yes						Yes	Yes	Yes	Yes	Yes	7
Murphy, 2019 [56]	Yes	Yes	Yes	Yes	Yes	Yes	Yes						7
Annan, 2017 [2]	Yes	Yes	Yes	Yes	Yes	Yes	Yes						7
Le Cossec, 2017 [13]	Yes	Yes	Yes	Yes	Yes	Yes	Yes						7
Paxton, 2015 [66]	Yes	Yes	Yes	Yes	Yes	Yes	Yes						7
Ravi, 2014 [70]	*Yes*	*Yes*	Yes	Yes	Yes	Yes	Yes						7
Baker, 2011 [3]	Yes	Yes	Yes	Yes	Yes	Yes	Yes						7
Camberlin, 2011 [8]	Yes	Yes	Yes	Yes	Yes	Yes	Yes						7
Huang, 2011 [27]	Yes	Yes	Yes	Yes	Can’t tell	Yes	Yes						6
Styron, 2011 [78]	*Yes*	*Yes*	Yes	Yes	No	Yes	Yes						6
Paterson, 2010 [65]	Yes	Yes	Yes	Yes	No	Yes	Yes						6
Losina, 2004 [42]	Yes	Yes	Yes	Yes	Yes	Yes	Yes						7
Kreder, 1997 [37]	Yes	Yes	Yes	Yes	Yes	Yes	Yes						7

### Surgeon volume threshold definitions

Of the 18 included studies, seven grouped surgeons into either low or high-volume surgeons, seven grouped surgeons into three categories (high-, moderate- or medium-, and low-volume), two grouped surgeons into four categories, and two grouped surgeons into five categories (Table 1). The threshold used to define high-volume surgeon varied widely from >4 to ≥115 THAs per year, with nine of the 18 included studies using a threshold ≥50 THAs per year to define high-volume surgeons (Appendix 2).Eleven studies did not provide an explanation for the threshold (or cut-off) value used to define high- vs. low-volume, while seven studies used either tertiles (n = 1), quartiles (n = 3), percentiles (n = 2), or receiver operating characteristic (ROC) curves (n = 1) to create each category.

In total, the 18 studies reported on 207,042 (23.3%) patients who underwent primary THA by low-volume surgeons, 377,279 (42.5%) by high-volume surgeons, and 211,740 (23.9%) by intermediate categories of surgeons (Table 3). Surgeon volume data was missing or unspecified for 91,657 (10.3%) patients. Individual cohort sizes ranged from 232 to 409,844 patients per study. Of the 18 studies, three [2, 13, 78] did not present outcomes of interest stratified by surgeon volume.

**Table 3:** Preoperative patient characteristics of the included articles

First author, year	Volume	Volume threshold per year	N patients	N females	%	Age	CCI (mean)	CCI 0	%	CCI 1	%	CCI 2	%	CCI 3	%	CCI 4	%	CCI ≥ 5	%
Thomas, 2024 [80]	High volume	173.7	567	297	(52%)	72 ± 10													
Thomas, 2024 [80]	Low volume	42.8	567	306	(54%)	73 ± 9													
Ruangsomboon, 2024 [72]	All		9672	5108	(53%)	*67 (median)*		7773	(80%)	997	(10.0%)	612	(6.3%)	150	(1.6%)	54	(0.6%)	86	(0.9%)
Ruangsomboon, 2024 [72]	High volume	>60	4399	2232	(51%)	*66 (median)*		3592	(82%)	428	(9.7%)	257	(5.8%)	70	(1.6%)	16	(0.4%)	36	(0.8%)
Ruangsomboon, 2024 [72]	Medium volume	30–60	2005	1075	(54%)	*68 (median)*		1606	(80%)	213	(11.0%)	134	(6.7%)	25	(1.2%)	8	(0.4%)	19	(0.9%)
Ruangsomboon, 2024 [72]	Low volume	<30	3268	1801	(55%)	*68 (median)*		2575	(79%)	356	(11.0%)	221	(6.8%)	55	(1.7%)	30	(0.9%)	31	(0.9%)
Rajahraman, 2023 [68]	High volume	150.5	272	155	(57%)	59 ± 10	2.4												
Rajahraman, 2023 [68]	Low volume	38.5	371	233	(60%)	60 ± 11	2.6												
Brodeur, 2022 [6]	High volume	≥115	29,031	16,446	(56%)	65 ± 11		19,642	(67.7%)	9389	(32.3%)								
Brodeur, 2022 [6]	Low volume	≤20	16,268	8847	(54%)	66 ± 11		9697	(59.6%)	6571	(40.4%)								
Chou, 2019 [11]	All		6367	3111	(49%)	18–49: (27%) 50–64: (36%) 65–79: (31%) >80: (7%)		4478	(70.3%)	1295	(20%)	594	(9.4%)						
Chou, 2019 [11]	High volume	≥15	3514		(53%)	18–49: (26%) 50–64: (36%) 65–79: (32%) >80: (6%)			(73%)		(19%)		(7.8%)						
Chou, 2019 [11]	Low volume	<15	2853		(44%)	18–49: (27%) 50–64: (35%) 65–79: (29%) >80: (9%)			(67%)		(22%)		(11%)						
Hanly, 2019 [22]	High volume	>25	4033																
Hanly, 2019 [22]	Medium volume	10–25	4568																
Hanly, 2019 [22]	Low volume	≤10	4450																
Hanly, 2019 [22]	No surgeon code		9905																
Murphy, 2019 [56]	Highest volume	>50	98,080																
Murphy, 2019 [56]	High volume	26–50	110,915																
Murphy, 2019 [56]	Moderate volume	11–25	112,670																
Murphy, 2019 [56]	Low volume	6–10	50,179																
Murphy, 2019 [56]	Lowest volume	1–5	38,000																
Annan, 2017 [2]	All		3744	2700	(72%)	68													
Le Cossec, 2017 [13]	All		62,906	36,045	(57%)	69 ± 11													
Le Cossec, 2017 [13]	High volume	>48	14,141	8190	(58%)	40–60: 2644 (19%) 60–75: 6673 (47%) ≥75: 4824 (34%)													
Le Cossec, 2017 [13]	Medium volume	18–48	45,348	25,897	(57%)	40–60: 8330 (18%) 60–75: 20,436 (45%) ≥75: 16,582 (37%)													
Le Cossec, 2017 [13]	Low volume	<18	3417	1958	(57%)	40–60: 703 (21%) 60–75: 1456 (43%) ≥75: 1258 (37%)													
Paxton, 2015 [66]	All		12,030	7093	(59%)	67 ± 11													
Paxton, 2015 [66]	High volume	≥50	3859																
Paxton, 2015 [66]	Medium volume	20–49	6425																
Paxton, 2015 [66]	Low volume	<20	1746																
Ravi, 2014 [70]	High volume	>35	6716	3716	(55%)	*70 (median)*		6119	(91%)	300	(4.5%)	297	(4.4%)						
Ravi, 2014 [70]	Low volume	<35	6716	3730	(56%)	*69 (median)*		6110	(91%)	302	(4.5%)	304	(4.5%)						
Baker, 2011 [3]	All		232	139		67													
Baker, 2011 [3]	High volume	>52	146	81	(55%)	64 ± 12													
Baker, 2011 [3]	Low volume	≤52	86	49	(57%)	71 ± 9													
Camberlin, 2011 [8]	All		11,856		(61%)	68 ± 12													
Camberlin, 2011 [8]	High volume	>20	9292		(60%)	67 ± 12	0.4												
Camberlin, 2011 [8]	Medium volume	7–20	1960		(62%)	69 ± 11	0.4												
Camberlin, 2011 [8]	Low volume	≤6	604		(59%)	67 ± 13	0.5												
Huang, 2011 [27]	High volume	8–119	6406	3030	(47%)	57 ± 15	0.23	5308	(83%)	863	(13.47%)	235	(3.67%)						
Huang, 2011 [27]	Medium volume	2–7.5	2478	949	(38%)	57 ± 15	0.26	2031	(82%)	325	(13%)	122	(4.92%)						
Huang, 2011 [27]	Low volume	0.5–1.5	451	163	(36%)	57 ± 15	0.32	363	(80%)	57	(12.64%)	31	(7%)						
Styron, 2011 [78]	Highest volume	≥53	28,572		(55%)	64 ± 13	0.34												
Styron, 2011 [78]	High volume	22–52	28,450		(59%)	65 ± 13	0.35												
Styron, 2011 [78]	Moderate volume	11–21	26,599		(59%)	67 ± 13	0.36												
Styron, 2011 [78]	Low volume	1–10	30,925		(60%)	68 ± 13	0.42												
Styron, 2011 [78]	Missing volume		78,008		(57%)	65 ± 13	0.37												
Paterson, 2010 [65]	All		20,290	11,660	(58%)	20–29: 108 (0.5%) 30–39: 362 (2%) 40–49: 1301 (6%) 50–59: 3038 (15%) 60–69: 5569 (27%) 70–79: 7227 (36%) 80–89: 2580 (13%) >90: 105 (0.5%)		1749	(9%)	629	(3%)	429	(2%)						
Paterson, 2010 [65]	Highest volume	>60	5018																
Paterson, 2010 [65]	High volume	41–60	4231																
Paterson, 2010 [65]	Moderate volume	26–40	6562																
Paterson, 2010 [65]	Low volume	1–25	4479																
Losina, 2004 [42]	All		57,488	36,845	(0.6%)	<75: (55%) >75: (45%)		≤1: 49,565 (86%)				>1:7923(14%)							
Losina, 2004 [42]	High volume	>12	15,106																
Losina, 2004 [42]	Low volume	<12	42,382																
Kreder, 1997 [37]	High volume	>10	4531		(59%)	67		(24.1%)				(3.4%)							
Kreder, 1997 [37]	Medium volume	2–10	3125		(59%)	68		(26.4%)				(3%)							
Kreder, 1997 [37]	Low volume	<2	280		(60%)	68		(34.3%)				(3.6%)							

### Revisions

**Fig. 2:**
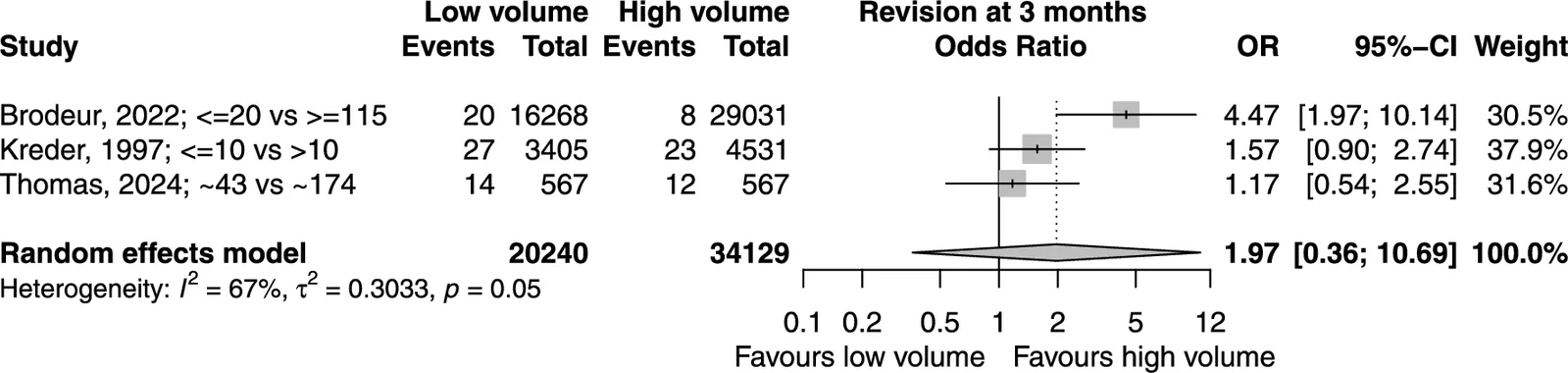
The pooled proportion of revision rates at 3 months was not significantly different between groups (OR = 2.0, 95%CI = 0.4–10.7; *p* = 0.228; three studies)

**Fig. 3:**
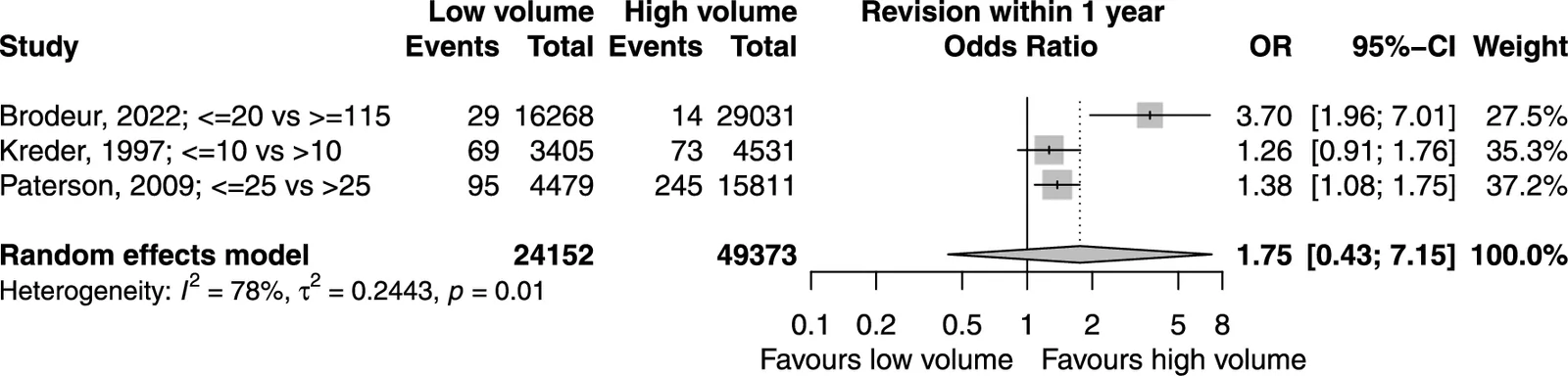
The pooled proportion of revision rates at 12 months was not significantly different between groups (OR = 1.8, 95%CI = 0.4–7.2, *p* = 0.228; three studies)

**Fig. 4:**
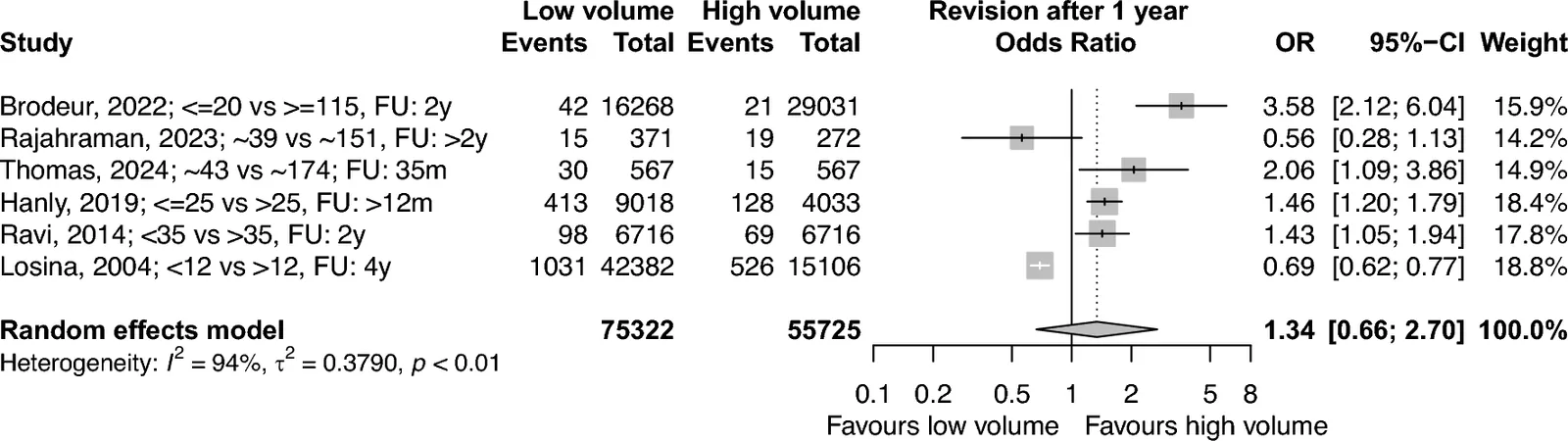
The pooled proportion of revision rates at >12 months was not significantly different between groups (OR = 1.3, 95%CI = 0.7–2.7; *p* = 0.334, six studies)

Revision rates were reported in eight studies [6, 22, 37, 42, 65, 68, 70, 80], at follow-ups of three to 48 months, and ranged from 0.1 to 5.3% for the low-volume group compared to <0.1 to 7.0% for the high-volume group (Table 4). The pooled proportion of revision rates was not significantly different between groups at 3 months (OR = 2.0, 95%CI = 0.4–10.7; *p* = 0.228; three studies) (Fig. 2), 12 months (OR = 1.8, 95%CI = 0.4–7.2; *p* = 0.228; three studies) (Fig. 3), or >12 months (OR = 1.3, 95%CI = 0.7–2.7; *p* = 0.334, six studies), although they tended to be lower for high-volume surgeons compared to low volume surgeons (Fig. 4). There was one study [13] that investigated the effect of surgeon volume as a continuous measure on revision rates, with multivariate regression analyses identifying a significant association between lower-volume surgeons and greater revision rates (hazards ratio adjusted for surgeon and hospital volume, demographic variables, and medical conditions [aHR] = 1.2 [95% CI = 1.1–1.3]).

**Table 4:** Outcomes reported in the included articles

First author	Group explanation	Volume definition	N patients	Follow-up (months)	All complications	Readmission	Revision	Mortality
Thomas, 2024 [80]	High volume	173.7	567	3 and 35		3 m: 24 (4.2%)	3 m: 12 (2.1%) 35 m: 15 (2.6%)	3 m: 12 (2.1%)
Thomas, 2024 [80]	Low volume	42.8	567	3 and 35		3 m: 41 (7.2%)	3 m: 14 (2.5%) 35 m: 30 (5.3%)	3 m: 19 (3.4%)
Ruangsomboon, 2024 [72]	High volume	>60	4399	1 and 12	12 m: 96 (2.2%)	1 m: 150 (3.4%)		12 m: 28 (0.6%)
Ruangsomboon, 2024 [72]	Medium volume	30–60	2005	1 and 12	12 m: 45 (2.2%)	1 m: 73 (3.6%)		12 m: 14 (0.7%)
Ruangsomboon, 2024 [72]	Low volume	<30	3268	1 and 12	12 m: 101 (3.1%)	1 m: 159 (4.9%)		12 m: 25 (0.8%)
Rajahraman, 2023 [68]	High volume	150.5	272	3 and >24	3 m: 15 (5.5%)	3 m: 22 (8.1%)	>24 m: 19 (7.0%)	
Rajahraman, 2023 [68]	Low volume	38.5	371	3 and >24	3 m: 16 (4.3%)	3 m: 17 (4.6%)	>24 m: 15 (4.0%)	
Brodeur, 2022 [6]	High volume	≥115	29,031	1, 3, 12, and 24	12 m: 4386 (15%)	1 m: 1135 (3.9%) 3 m: 1737 (6%)	3 m: 8 (<0.1%) 12 m: 14 (0.1%) 24 m: 21 (0.1%)	12 m: 79 (0.3%)
Brodeur, 2022 [6]	Low volume	≤20	16,268	1, 3, 12, and 24	12 m: 4441 (27%)	1 m: 1139 (7%) 3 m: 1661 (10.2%)	3 m: 20 (0.1%) 12 m: 29 (0.2%) 24 m: 42 (0.3%)	12 m: 295 (0.7%)
Chou, 2019 [11]	High volume	≥15	3514	1		1 m: 113 (3.2%)		
Chou, 2019 [11]	Low volume	<15	2853	1		1 m: 168 (5.9%)		
Hanly, 2019 [22]	High volume	>25	4033	>12			>12 m: 128 (3.2%)	
Hanly, 2019 [22]	Medium volume	10–25	4568	>12			>12 m: 180 (3.9%)	
Hanly, 2019 [22]	Low volume	≤10	4450	>12			>12 m: 233 (5.2%)	
Hanly, 2019 [22]	No surgeon code		9905	>12			>12 m: 628 (6.3%)	
Murphy, 2019 [56]	Highest volume	>50	98,080	3		3 m: 5885 (6.0%)		3 m: 235 (0.2%)
Murphy, 2019 [56]	High volume	26–50	1,10,915	3		3 m: 7653 (6.9%)		3 m: 410 (0.4%)
Murphy, 2019 [56]	Moderate volume	11–25	1,12,670	3		3 m: 9126 (8.1%)		3 m: 586 (0.5%)
Murphy, 2019 [56]	Low volume	6–10	50,179	3		3 m: 4616 (9.2%)		3 m: 366 (0.7%)
Murphy, 2019 [56]	Lowest volume	1–5	38,000	3		3 m: 4066 (10.7%)		3 m: 483 (1.3%)
Paxton, 2015 [66]	High volume	≥50	3859	1		1 m: 163 (4.2%)		
Paxton, 2015 [66]	Medium volume	20–49	6425	1		1 m: 203 (3.2%)		
Paxton, 2015 [66]	Low volume	<20	1746	1		1 m: 70 (4%)		
Ravi, 2014 [70]	High volume	>35	6716	3 and 24	24 m: 273 (4.1%)		24 m: 69 (1.0%)	3 m: 30 (0.5%)
Ravi, 2014 [70]	Low volume	<35	6716	3 and 24	24 m: 330 (4.9%)		24 m: 98 (1.5%)	3 m: 44 (0.7%)
Camberlin, 2011 [8]	High volume	>20	9292	3	3 m: 276 (2.9%)			
Camberlin, 2011 [8]	Medium volume	≤20	1960	3	3 m: 89 (4.5%)			
Camberlin, 2011 [8]	Low volume	≤6	604	3	3 m: 30 (4.9%)			
Huang, 2011 [27]	High volume	8–119	6406	Periop	Periop: 86 (1.34%)			
Huang, 2011 [27]	Medium volume	2–7.5	2478	Periop	Periop: 47 (1.90%)			
Huang, 2011 [27]	Low volume	0.5–1.5	451	Periop	Periop: 8 (1.77%)			
Paterson, 2010 [65]	Highest volume	>60	5018	12	In-hospital: 330		12 m: 74 (1.5%)	3 m: 29 (<0.1%)
Paterson, 2010 [65]	High volume	41–60	4231	12	In-hospital: 236		12 m: 73 (1.7%)	3 m: 24 (<0.1%)
Paterson, 2010 [65]	Moderate volume	26–40	6562	12	In-hospital: 329		12 m: 98 (1.5%)	3 m: 36 (<0.1%)
Paterson, 2010 [65]	Low volume	1–25	4479	12	In-hospital: 313		12 m: 95 (2.1%)	3 m: 33 (<0.1%)
Losina, 2004 [42]	High volume	>12	15,106	48			48 m, HV > 100: 137 (3.3%) 48 m, HV 51–100: 215 (3.3%) 48 m, HV 26–50: 136 (3.9%) 48 m, HV 1–25: 38 (4.0%)	
Losina, 2004 [42]	Low volume	<12	42,382	48			48 m, HV > 100: 81 (4.9%) 48 m, HV 51–100: 361 (4.4%) 48 m, HV 26–50: 113 (4.0%) 48 m, HV 1–25: 476 (4.9%)	
Kreder, 1997 [37]	High volume	>10	4531	3 and 12	In-hospital: 399 (8.8%)		3 m: 23 (0.5%) 12 m: 73 (1.6%)	3 m: 23 (0.5%) 12 m: 59 (1.3%)
Kreder, 1997 [37]	Medium volume	2–10	3125	3 and 12	In-hospital: 247 (7.9%)		3 m: 22 (0.7%) 12 m: 60 (1.9%)	3 m: 28 (0.9%) 12 m: 63 (2.0%)
Kreder, 1997 [37]	Low volume	<2	280	3 and 12	In-hospital: 36 (12.9%)		3 m: 5 (1.8%) 12 m: 9 (3.2%)	3 m: 6 (2.1%) 12 m: 9 (3.2%)

*N* number, *FU* follow-up, *HV* hospital volume

### Complications

Complication rates were reported in eight studies [6, 8, 27, 37, 65, 68, 70, 72] with follow-up times ranging from perioperative timepoint to 24 months postoperatively. Overall, the rates were similar for the low- vs. high-volume groups (Table 4). Complications reported across studies included fractures [70, 72], dislocations [8, 68, 70, 72], deep and superficial infections or inflammation [6, 8, 65, 68, 70, 72], sepsis [68], venous thromboembolism and vascular complications [6, 8, 27, 65, 68, 70], mechanical complications [8, 65], acute myocardial infarctions [6, 27, 37], strokes [6, 37], pneumonia and respiratory complications [6, 27, 65]. Furthermore, the definition of complications varied across studies (Appendix 4), with some studies counting the complications that lead to revision, readmission, or in-hospital mortality [6, 70, 72]. The pooled proportion of complication rates was not calculated since the follow-up time and definition of complications varied greatly across studies.

Dislocation rates were reported in three studies [68, 70, 72], at follow-ups of three, 12, and 24 months, and were 0.5, 0.4, and 1.9% for the low-volume group compared to 0, 0.3, and 1.3% for the high-volume group (Appendix 4). The pooled proportion of dislocation rates could not be calculated due to the great variability of follow-up times across studies. There was one study [2] that investigated the effect of surgeon volume as a continuous measure on dislocation rates, with multivariate regression analyses identifying a significant association between lower-volume surgeons and greater dislocation rates (OR = 0.97; *p* < 0.001).

**Fig. 5:**
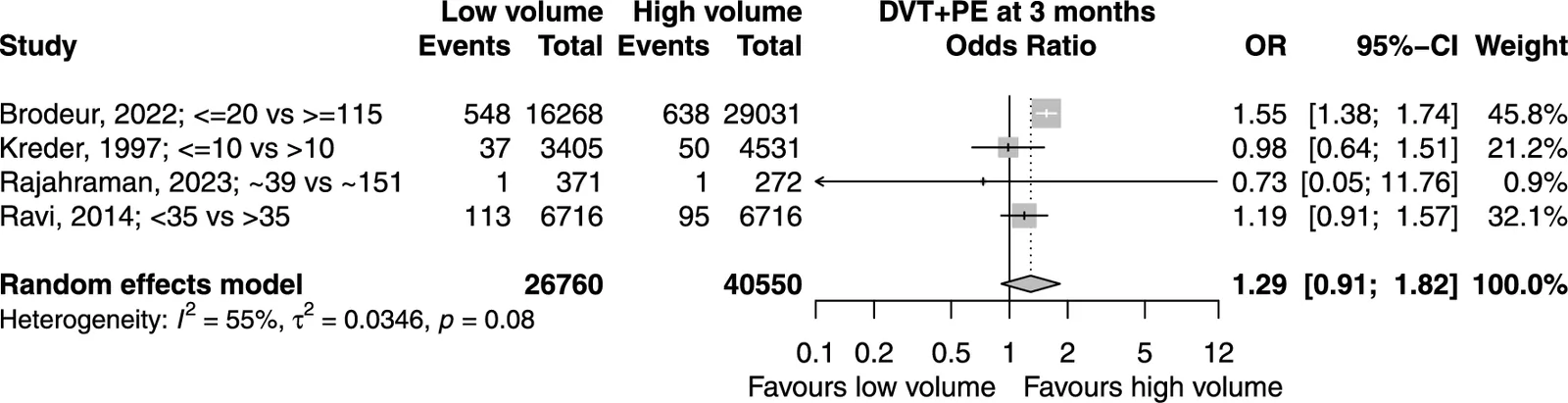
The pooled proportion of venous thromboembolism rates at 3 months was not significantly different between groups (OR = 1.3, 95%CI = 0.9–1.8; *p* = 0.104, four studies)

Venous thromboembolism rates (including deep vein thrombosis and/or pulmonary embolism) were reported in four studies [6, 37, 68, 70], at follow-ups of 3–12 months, and ranged from 0.3 to 3.4% for the low-volume group compared to 0.4 to 2.2% for the high-volume group (Appendix 4). The pooled proportion of short-term venous thromboembolism rates was not significantly different between groups (OR = 1.3, 95%CI = 0.9–1.8; *p* = 0.104, four studies), although it tended to be lower for high-volume surgeons compared to low-volume surgeons (Fig. 5). The pooled proportion of mid-term venous thromboembolism rates was not calculated since only one study reported this variable.

Transfusion rates were reported in one study [3], and were 17% for the low-volume group compared to 5% for the high-volume group at 1 month (Appendix 4), which was reported to be statistically significant (risk ratio [RR] = 3.3, 95%CI = 1.5–9.1; *p* = 0.003).

### Readmissions

**Fig. 6:**
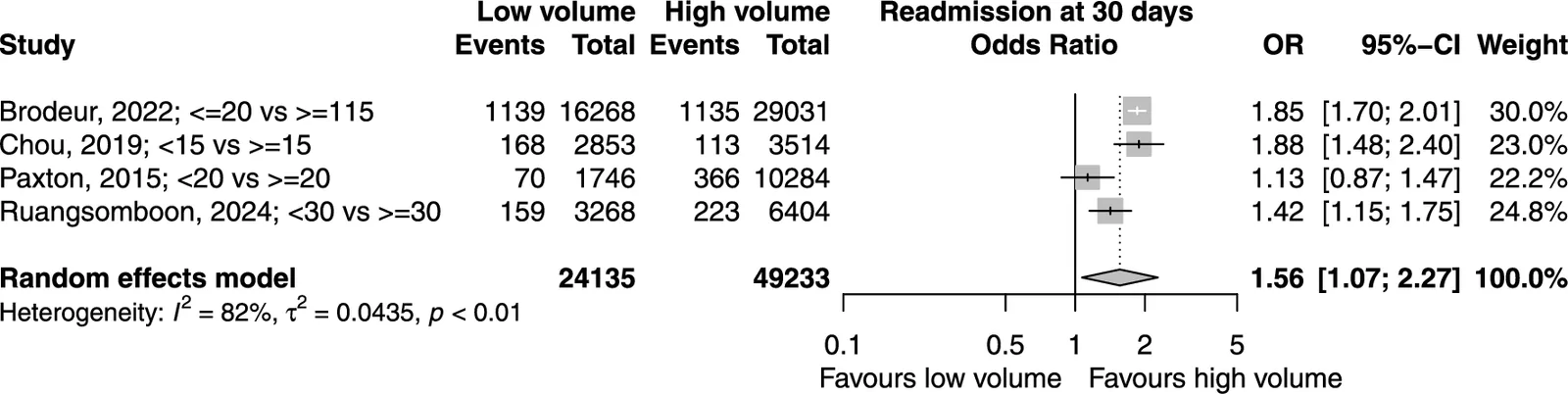
The pooled proportion of readmission rates at 1 month was significantly lower for high-volume surgeons compared to low-volume surgeons (OR = 1.6, 95%CI = 1.1–2.3; *p* = 0.033, four studies)

**Fig. 7:**
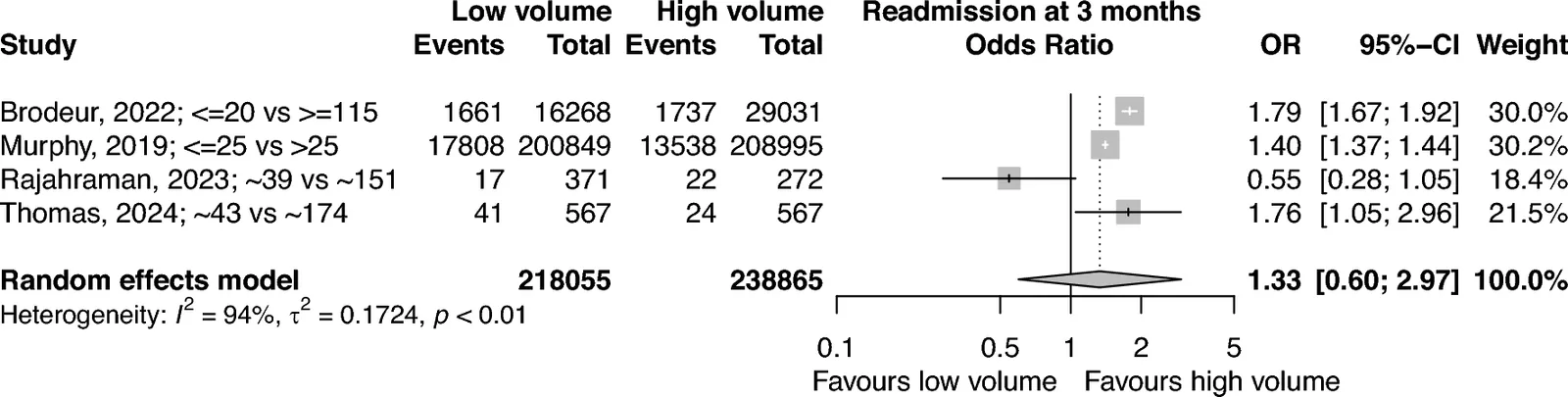
The pooled proportion of readmission rates at 3 months was not significantly different between groups (OR = 1.3, 95%CI = 0.6–3.0; *p* = 0.337, four studies)

Readmission rates were reported in seven studies [6, 11, 56, 66, 68, 72, 80], at follow-ups of 1–3 months, and ranged from 4.0 to 10.2% for the low-volume group compared to 3.2 to 8.1% for the high-volume group (Table 4). The pooled proportion of one month readmission rates was significantly lower for high-volume surgeons compared to low-volume surgeons (OR = 1.6, 95%CI = 1.1–2.3; *p* = 0.033, four studies) (Fig. 6), while there were no significant differences in the pooled proportion of 3 month readmission rates between groups (OR = 1.3, 95%CI = 0.6–3.0; *p* = 0.337, four studies), although it tended to be lower for high-volume surgeons compared to low-volume surgeons (Fig. 7).

### Mortality

**Fig. 8:**
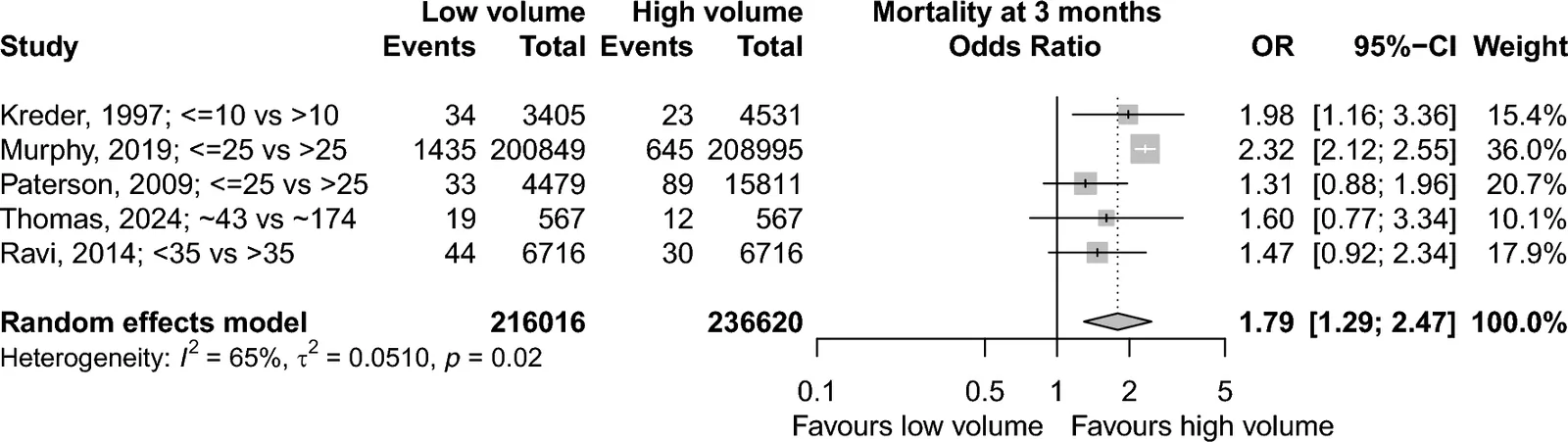
The pooled proportion of mortality rates at 3 months was significantly lower for high-volume surgeons compared to low-volume surgeons (OR = 1.8, 95%CI = 1.3–2.5; *p* = 0.008, five studies)

**Fig. 9:**
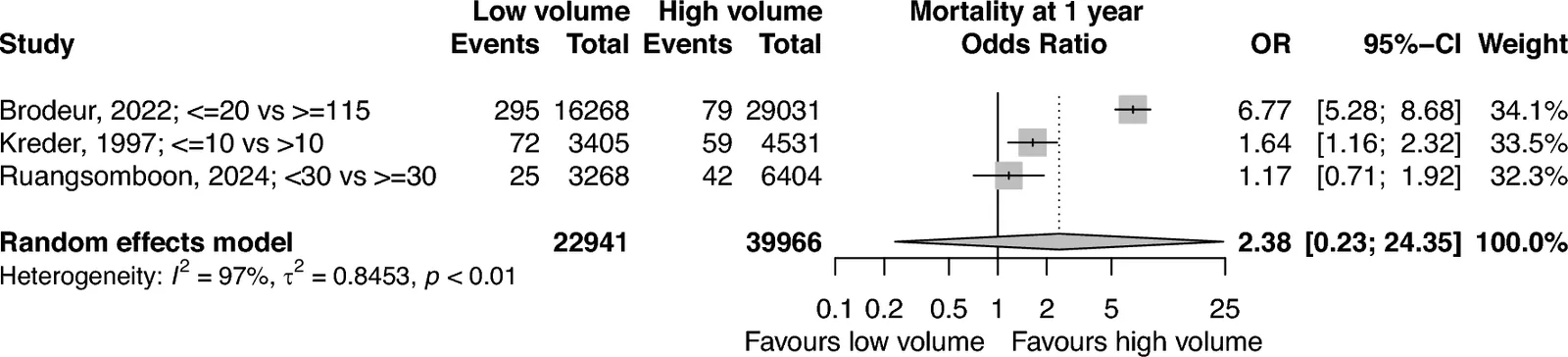
The pooled proportion of mortality rates at 12 month was not significantly different between groups (OR = 2.4, 95%CI = 0.23–24.4; *p* = 0.249, 3 months)

Mortality rates were reported in seven studies [6, 37, 56, 65, 70, 72, 80], at follow-ups of 3–12 months, and ranged from <0.1 to 3.4% for the low-volume group compared to <0.1 to 2.1% for the high-volume group (Table 4). The pooled proportion of 3 month mortality rates was significantly lower for high-volume surgeons compared to low-volume surgeons (OR = 1.8, 95%CI = 1.3–2.5; *p* = 0.008, five studies) (Fig. 8), while there were no significant differences in the pooled proportion of 12 month mortality rates between groups (OR = 2.4, 95%CI = 0.23–24.4; *p* = 0.249, 3 months), although it tended to be lower for high-volume surgeons compared to low-volume surgeons (Fig. 9).

### Clinical outcomes

One of the 18 studies [68] reported clinical outcomes stratified by surgeon volume. At 12 months, Hip Disability and Osteoarthritis Outcome Score (HOOS) was 70.4 ± 16.7 for the low-volume group compared to 78.2 ± 18.1 for the high-volume group (*p* = 0.047). At 24 months, Forgotten Joint Score (FJS) was 71.0 ± 24.9 for the low-volume group compared to 64.0 ± 33.1 for the high-volume group (*p* = 0.485).

## Discussion

The included studies in this systematic review and meta-analysis demonstrated large variability in surgeon volume definitions. There remains a trend suggesting that higher-volume surgeons have fewer revisions, complications, readmissions, and mortality compared to lower-volume surgeons.

The present systematic review identified large variations in surgeon volume definitions, with thresholds ranging from eight to 173 THAs per year for high-volume definition. While some of the studies did not justify the choice of cutoff value, others used tertiles or quartiles to determine pragmatic thresholds. Of the 18 included studies, nine used a threshold ≥50 THAs per year to define high-volume surgeons. The variability in surgeon volume definitions across studies highlights the need for a standardized threshold for future studies to facilitate volume-outcome investigations. Surgeons with more cases per year are more likely to implant the cup and stem correctly, have shorter operating times, and lower risks of complications and revisions [82].

The association between surgeon volume and postoperative outcomes following primary THA are consistent with the literature. A previous systematic review published in 2018 by Malik et al. [44], investigating the effect of surgeon volume on outcomes following hip fracture, reported that lower-volume surgeons had greater mortality rates. However, results were inconsistent across included studies in terms of postoperative complications, as only the increased risk of transfusion in the low-volume group was statistically significant. While patient characteristics slightly differed from the present review, the findings of Malik et al. supported a noticeable trend in the effect of surgeon volume following hip surgery, implying that technical experience can contribute to improved patient safety. The review by Malik et al. [44] reported on patients with hip fractures undergoing either open reduction and internal fixation, hemiarthroplasty, or THA; while the present review focused only on patients undergoing primary THA. Due to the differences in indication and surgery, there was no overlap in included studies. Furthermore, Malik et al. [44] included only five studies reporting on surgeon volume, while the present review included 18 studies, allowing for a more robust investigation on the effects of surgeon volume.

Interestingly, transfusion rates in the present review (5–17%) exceeded those typically reported in other THA studies (1.2–2.5%) [50, 51]. Similarly, 90-day mortality rates in the present review (low-volume, <0.1–3.4%; high-volume, <0.1–2.1%) appear high compared to modern practice, where rates typically remain below 2% [17]. Despite the range in mortality rates, there remains a consistent trend showing lower mortality in patients treated by high-volume surgeons, suggesting that more experience leads to improved safety and outcomes. The differences in ranges across studies may be explained by variations in patient demographics and comorbidities, case complexity, care-pathways, surgical protocols, geographical location, and use of registries vs. hospital databases among others. These findings highlight the importance of considering context when interpreting results.

A systematic review by Mufarrih et al. [55] investigating the effect of hospital volume on THA outcomes reported that low-volume hospitals had higher infection rates, and greater short- to mid-term mortality. In practice, high-volume surgeons are more likely to operate in high-volume or specialized centers. As discussed by Mufarrih et al. [55], these infrastructures can offer fast-response, standardized perioperative pathways, and multidisciplinary teams which may also play a major role in postoperative outcomes. Furthermore, patient-specific characteristics such as BMI, comorbidities, ASA score, and socio-economic status may affect complications and increase recovery time [1, 73]. Therefore, the differences observed in the present study between high- and low-volume surgeons, may in fact be explained by a combination of patient-, surgeon-, and hospital-specific factors. High volume surgeons are more likely working at high volume hospitals, with dedicated arthroplasty units, and enhanced recovery after surgery (ERAS) pathways. To be able to study the effect of surgeon volume on outcomes in isolation, it is necessary for studies to adjust for these potential confounding factors.

This study has several limitations. First, all included studies were retrospective in design, which limits the level of evidence and may increase the susceptibility to bias. Second, there was considerable heterogeneity across included studies regarding surgeon volume definition, making it difficult to identify trends and draw definitive conclusions. Third, most studies did not report on clinical scores, preventing further analysis on patient function and quality of life. Finally, the follow-up duration and the definition of complications varied greatly across studies. To account for the effect of follow-up on outcomes, the data was stratified into different follow-up groups.

## Conclusions

In conclusion, the included studies demonstrated considerable variability in the definitions of surgeon volume. However, there remains a trend suggesting that higher-volume surgeons experience fewer revisions, complications, readmissions, and mortality; although these differences were largely not statistically significant. Clinicians should note that surgeon volume may not be the only factor influencing outcomes, as hospital and patient characteristics may be associated with short- to long-term outcomes following primary THA.

## Supplementary Information

Below is the link to the electronic supplementary material.

Supplementary file1 (DOCX 12 KB)

Supplementary file2 (DOCX 67 KB)

Supplementary file3 (XLSX 78 KB)

Supplementary file4 (XLSX 104 KB)

Supplementary file5 (DOCX 31 KB)

## Data Availability

Data extracted from included studies and used for statistical analyses, as well as the statistical code, are available from the corresponding author upon reasonable request.
