# Supplementary material for: The effect of surgeon volume on outcomes after total hip arthroplasty: a systematic review and meta-analysis
Source: Arch Orthop Trauma Surg. 2026 Aug 20;146(1):308. doi: 10.1007/s00402-026-06434-w (PMC13493433; doi:10.1007/s00402-026-06434-w)
Supplement: Supplementary file 1 [file 402_2026_6434_MOESM1_ESM.docx]

**Search strategy**

#1 hip AND (arthroplasty OR replacement)

#2 "surgeon volume" OR "provider volume" OR "procedure volume" OR "volume relationship" OR "high volume" OR "low volume" OR "volume-outcome"

#1 AND #2
