# Supplementary material for: The effect of surgeon volume on outcomes after total hip arthroplasty: a systematic review and meta-analysis
Source: Arch Orthop Trauma Surg. 2026 Aug 20;146(1):308. doi: 10.1007/s00402-026-06434-w (PMC13493433; doi:10.1007/s00402-026-06434-w)
Supplement: Supplementary file 2 [file 402_2026_6434_MOESM2_ESM.docx]

***Sensitivity analysis including studies that defined high-volume surgeons as ≥50 THAs per year***

Of the 18 included articles, 9 used a threshold ≥50 to define high-volume surgeons [1-9] (Table 4). The pooled proportion of revision rates was not significantly different between groups at three months (OR=2.3, 95%CI=0.0–11215.4; p=0.436, two studies), 12 months (OR=2.0, 95%CI=0.0–2759.9; p=0.434, two studies), or >12 months (OR=1.6, 95%CI=0.16–17.2; p=0.464, three studies). The pooled proportion of short-term venous thromboembolism rates was significantly lower for high-volume surgeons compared to low-volume surgeons (OR=1.3, 95%CI=0.9–1.8; p=0.045, two studies). The pooled proportion of readmission rates was not significantly different between groups at one month (OR=1.2, 95%CI=0.42–3.7; p=0.475, three studies) or three months (OR=1.3, 95%CI=0.60–3.0; p=0. 340, four studies). The pooled proportion of mortality rates was not significantly different between groups at three months (OR=1.7, 95%CI=0.54–5.1; p=0.192, three studies) or 12 months (OR=2.8, 95%CI=0.00–205227; p=0.446, two studies). Although there were no significant differences between groups for most outcomes, there was a trend for high-volume surgeons to provide better outcomes compared to low-volume surgeons [1-7, 9].

**References**

[1] Baker P, Dowen D, McMurtry I. The effect of surgeon volume on the need for transfusion following primary unilateral hip and knee arthroplasty. Surgeon 2011;9:1:13-7 <https://doi.org/http://dx.doi.org/10.1016/j.surge.2010.08.011>.

[2] Brodeur PG, Boduch A, Kim KW, Cohen EM, Gil JA, Cruz AI, Jr. Surgeon and facility volumes are associated with social disparities and post-operative complications after total hip arthroplasty. Journal of Arthroplasty 2022;37:8:S908-S18.e1 <https://doi.org/http://dx.doi.org/10.1016/j.arth.2022.02.018>.

[3] Murphy WS, Cheng T, Lin B, Terry D, Murphy SB. Higher volume surgeons have lower medicare payments, readmissions, and mortality after tha. Clinical orthopaedics and related research 2019;477:2:334-41 <https://doi.org/http://dx.doi.org/10.1097/CORR.0000000000000370>.

[4] Paterson JM, Williams JI, Kreder HJ, Mahomed NN, Gunraj N, Wang X et al. Provider volumes and early outcomes of primary total joint replacement in ontario. Canadian journal of surgery Journal canadien de chirurgie 2010;53:3:175-83.

[5] Paxton EW, Inacio MCS, Singh JA, Love R, Bini SA, Namba RS. Are there modifiable risk factors for hospital readmission after total hip arthroplasty in a us healthcare system? Clinical orthopaedics and related research 2015;473:11:3446-55 <https://doi.org/http://dx.doi.org/10.1007/s11999-015-4278-x>.

[6] Rajahraman V, Shichman I, Berzolla E, Rozell J, Meftah M, Schwarzkopf R. Are patient outcomes affected by surgeon experience with total hip arthroplasty in morbidly obese patients? Arthroplasty Today 2023;23 <https://doi.org/http://dx.doi.org/10.1016/j.artd.2023.101207>.

[7] Ruangsomboon P, Bagouri E, Pincus D, Paterson JM, Ravi B. Association of surgeon volume with complications following direct anterior approach (daa) total hip arthroplasty: A population-based study. Acta Orthop 2024;95:505-11 <https://doi.org/10.2340/17453674.2024.41506>.

[8] Styron JF, Koroukian SM, Klika AK, Barsoum WK. Patient vs provider characteristics impacting hospital lengths of stay after total knee or hip arthroplasty. Journal of Arthroplasty 2011;26:8:1418-26 <https://doi.org/http://dx.doi.org/10.1016/j.arth.2010.11.008>.

[9] Thomas J, Ashkenazi I, Lawrence KW, Davidovitch RI, Rozell JC, Schwarzkopf R. Should high-risk patients seek out care from high-volume surgeons? The bone & joint journal 2024;106-B:3 Supple A:10-6 <https://doi.org/http://dx.doi.org/10.1302/0301-620X.106B3.BJJ-2023-0807.R1>.
